# Supplementary material for: IQGAP1 Regulates Actin Polymerization and Contributes to Bleomycin-Induced Lung Fibrosis
Source: Int J Mol Sci. 2024 May 11;25(10):5244. doi: 10.3390/ijms25105244 (PMC11121427; doi:10.3390/ijms25105244)
Supplement: Supplementary file 1 [file ijms-25-05244-s001.zip › ijms-2940834-supplementary.pdf]

Supplementary Materials

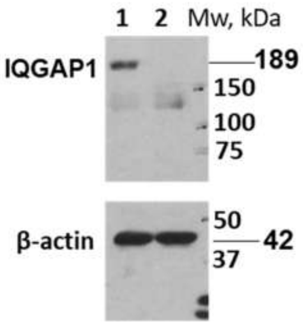

Supplementary Figure S1. IQGAP1 is expressed in lung homogenates of wild type mouse (lane 1) but not in IQGAP1-KO mouse (lane 2). Lyophilized lung tissue was solubilized in buffer containing 50mM Tris, pH 7.4; 10mM EDTA; 150mM NaCl; 1% Nonidet P-40; 0.5% deoxycholate; 0.1% SDS, and protease inhibitor cocktail. Protein concentration was determined with BCA™ protein assay kit (Pierce, Rockford, IL), and 40 micrograms of protein per sample was resolved on 4-20% gradient SDS-PAGE.

Following the separation, the proteins were electrophoretically transferred to a nitrocellulose membrane. The upper part of the membrane was analyzed by Western blot using anti-IQGAP1 antibody from Santa Cruz Biotechnology (Santa Cruz, CA) and lower part of the membrane was immunoblotted with anti-β-actin antibody from Sigma-Aldrich (St. Louis, MO), used as a loading control.

Table S1. List of Primers Used for Quantitative Real-Time PCR

Gene	Forward primer	Reverse primer	Tm (°C)
Col1a1	GCCAAGAAGACATCCCTGAAG	GTGGCAGATACAGATCAAGC	59
Acta2	CGAAACCACCTATAACAGCATCA	GCGTTCTGGAGGGGCAAT	61
Tgfβ1	GACTCTCCACCTGCAAGACC	GGACTGGCGAGCCTTAGTTT	62
Tnfα	AAGCCTGTAGCCCACGTCGTA	AGGTACAACCCATCGGCTGG	64
Il6	TCCATCCAGTTGCCTTCTTG	TTCCACGATTTCCCAGAGAAC	61
Gapdh	GGGTCCCAGCTTAGGTTCAT	TACGGC CAAATCCGTTCACA	61
